# Construction of hollow polydopamine nanoparticle based drug sustainable release system and its application in bone regeneration

**DOI:** 10.1038/s41368-021-00132-6

**Published:** 2021-08-18

**Authors:** Lu Wang, Shuwei Liu, Chunxia Ren, Siyuan Xiang, Daowei Li, Xinqing Hao, Shilei Ni, Yixin Chen, Kai Zhang, Hongchen Sun

**Affiliations:** 1 Department of Oral Pathology, Hospital of Stomatology, Jilin University, Changchun, China; 2 Jilin Provincial Key Laboratory of Tooth Development and Bone Remodeling, School and Hospital of Stomatology, Jilin University, Changchun, China; 3 State Key Laboratory of Supramolecular Structure and Materials, College of Chemistry, Jilin University, Changchun, China

**Keywords:** Drug delivery, Nanoparticles, Biomineralization

## Abstract

Nanomaterial-based drug sustainable release systems have been tentatively applied to bone regeneration. They, however, still face disadvantages of high toxicity, low biocompatibility, and low drug-load capacity. In view of the low toxicity and high biocompatibility of polymer nanomaterials and the excellent load capacity of hollow nanomaterials with high specific surface area, we evaluated the hollow polydopamine nanoparticles (HPDA NPs), in order to find an optimal system to effectively deliver the osteogenic drugs to improve treatment of bone defect. Data demonstrated that the HPDA NPs synthesized herein could efficiently load four types of osteogenic drugs and the drugs can effectively release from the HPDA NPs for a relatively longer time in vitro and in vivo with low toxicity and high biocompatibility. Results of qRT-PCR, ALP, and alizarin red S staining showed that drugs released from the HPDA NPs could promote osteogenic differentiation and proliferation of rat bone marrow mesenchymal stem cells (rBMSCs) in vitro. Image data from micro-CT and H&E staining showed that all four osteogenic drugs released from the HPDA NPs effectively promoted bone regeneration in the defect of tooth extraction fossa in vivo, especially tacrolimus. These results suggest that the HPDA NPs, the biodegradable hollow polymer nanoparticles with high drug load rate and sustainable release ability, have good prospect to treat the bone defect in future clinical practice.

## Introduction

In recent years, nanomaterials have been more and more widely used in biomedicine with the development of nanotechnology,^1–3^ and nanomaterial-based drug sustainable release systems have been well developed.^4–7^ The nanomaterial-based drug sustainable release systems have been widely used not only in tumor therapy but also in tissue regeneration and in many other fields.^8–10^ The sustainable release can enhance the drugs to retain their effects for a long time, avoid being rapidly metabolized, and ensure the efficacy of drugs.^11,12^ At the same time, local drug release can specifically increase drug concentration at the lesion site and decrease the side effects compared to other conventional deliveries.^13,14^ Various drug sustainable release systems provide a novel means to effectively treat diseases.^15,16^

An ideal nanomaterial-based drug sustainable release system should have higher capacity of drug load, and efficiently and slowly release drug to the target site.^17,18^ External stimuli, such as laser, ultrasound, and magnetic field;^19^ specific physical and chemical properties at target site, such as pH changes and glutathione content; and physiological environment of target site, can all affect drug release.^20,21^ Furthermore, nanomaterials in drug sustainable release systems should have low toxicity and high biocompatibility, and it is better to be metabolizable and biodegradable in vivo.^22,23^ Currently, only few nanomaterials can meet all requirements. Therefore, there are limitations to construct an ideal drug sustainable release system. More rigorous design is needed to create an optimal drug sustainable release system.

Improving the surface area of nanomaterials is helpful to enhance the drug-loading capacity.^24,25^ In this regard, hollow nanomaterials have significant advantages, such as large specific surface area, low density, and high loading capacity.^26,27^ Among the hollow nanoparticles (NPs), hollow polymer NPs are widely concerned because of their lower toxicity, higher biocompatibility, and higher degradation ability.^28^ The representative one is polydopamine NPs.^29^ As one of the main pigment components in natural melanin, polydopamine is safe and has many functional groups integrated in its chemical structure, which provides many possibilities to construct nanocomposites.^30,31^ Drug molecules with ring structure can interact with the ring structure in polydopamine by *π*–*π* conjugation to realize the drug loading, then sustainable drug release can be achieved by the gradual degradation of polydopamine under physiological condition.^32^

Bone regeneration is a critical process for any bone defect.^33^ Many drugs can promote bone regeneration through different mechanisms.^34,35^ Aspirin is a nonsteroidal anti-inflammatory drug and can also inhibit osteoclast differentiation.^36^ Ascorbic acid can promote the formation of mineralized nodules and directly inhibit osteoclast formation induced by receptor activator of nuclear factor κB ligand (RANKL).^37^ Tacrolimus can induce the proliferation and differentiation of bone marrow mesenchymal stem cells (BMSCs) into osteoblastic cells.^38^ Another one is simvastatin, which can stimulate bone morphogenetic protein and promote the production of type I collagen (Col I).^39^ The problem is whether we can effectively and efficiently deliver the interesting drug to the bone defect area and retain relative long-term effect.^40,41^ Interestingly, the drug sustainable release systems maybe an excellent delivery method to promote bone regeneration.

**Fig. 1:**
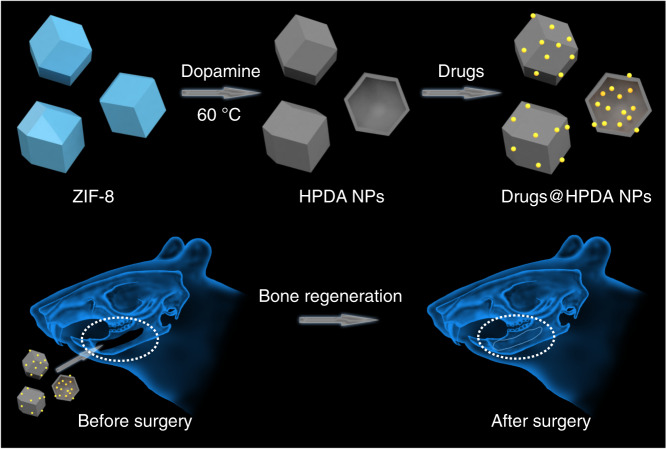
Schematic diagram of the construction of drug sustainable release system and its application in bone regeneration

To develop an efficient drug sustainable release system for bone regeneration, in the present study we synthesized hollow polydopamine NPs (HPDA NPs) using ZIF-8 and dopamine by means of chelation competition induced polymerization (CCIP). Interestingly, due to *π*–*π* conjugation interaction between nanomaterials with drug molecules, HPDA NPs were able to load four different bone regeneration drugs, which could efficiently release slowly, resulting in the osteogenic differentiation and bone regeneration in vitro and in vivo (Fig. 1). This design well solved the problem of bone loss caused by the absorption of alveolar ridge after tooth extraction, provided enough bone mass for the later denture repair and implant implantation, and has great potential application in the clinical practice.

## Results

**Fig. 2:**
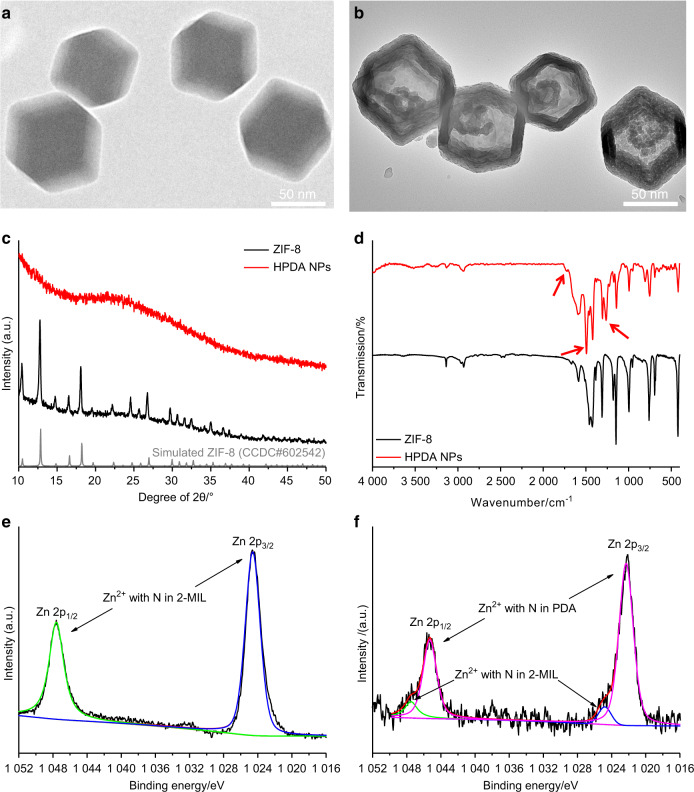
Physical characteristics of HPDA NPs. **a** TEM image of ZIF-8. **b** TEM image of HPDA NPs. **c** XRD patterns of ZIF-8 and HPDA NPs. **d** Infrared spectra of ZIF-8 and HPDA NPs. **e** XPS pattern of ZIF-8. **f** XPS pattern of HPDA NPs

ZIF-8 was synthesized by 2-methylimidazole (2-MIL) and zinc ions. Image from transmission electron microscopy (TEM) showed that the ZIF-8 was regular dodecahedral structure with an average diameter of 82.2 nm (Fig. 2a). Chemically, the coordination ability between dopamine monomers and zinc ions is stronger than that between 2-MIL and zinc ions.^29^ Therefore, when the dopamine monomers were added into the ZIF-8, the original 2-MIL on the surface of ZIF-8 was gradually replaced by the dopamine monomers on account of coordination competition. Dopamine monomers can polymerize in this weakly alkaline mixture to form polydopamine on the surface of ZIF-8; with the further disintegration of ZIF-8, polydopamine NPs with hollow structure were obtained. In general, this process was based on the chelation competition induced polymerization (CCIP) and HPDA NPs were synthesized with the breakdown of ZIF-8 and the polymerization of dopamine monomers. TEM image clearly showed the hollow structure of HPDA NPs. Average diameter of the HPDA NPs was 87.8 nm and average shell thickness was 16.4 nm (Fig. 2b). X-ray diffraction (XRD) analysis revealed that position and intensity of diffraction peaks of ZIF-8 correspond well to standard peaks, which was consistent with its crystal structure. These diffraction peaks, however, did not appear in the XRD pattern of HPDA NPs, which indicated that the crystal structure was destroyed. It was also consistent with the decomposition of ZIF-8 (Fig. 2c). Further, data from Fourier transform infrared spectroscopy (FTIR) also indicated that the HPDA NPs had their own specific peaks, whereas the ZIF-8 did not have. These characteristic peaks are marked with red arrows. Concretely, the peak at 1725 cm^−1^ corresponded to the stretching vibration peak of C=O, the peak at 1494 cm^−1^ corresponded to the skeleton vibration of the benzene ring, and the peak at 1260 cm^−1^ corresponded to the in-plane bending vibration peak of –OH, which coincides with the structure of polydopamine (Fig. 2d). Therefore, data from FTIR had confirmed the formation of HPDA NPs, which was also verified by X-ray photoelectron spectroscopy (XPS). In the XPS spectra of ZIF-8, the binding energy peaks of Zn corresponded to the coordination between Zn^2+^ and N in 2-MIL, which was consistent with the structure of ZIF-8 (Fig. 2e). In the XPS spectrum of HPDA NPs, the intensity of the coordination peak of Zn^2+^ and N in 2-MIL decreased significantly, whereas at the slightly lower binding energy, two new high strength peaks appeared, corresponding to the coordination of Zn^2+^ and N in HPDA NPs (Fig. 2f). The pore structure and specific surface area of HPDA NPs were investigated by N_2_ sorption method. The N_2_ adsorption/desorption isotherm of HPDA NPs exhibited an obviously hysteresis loop, corresponding to the mesoporous structure (Supplementary Fig. S1a). Further, the pore diameter was mainly concentrated in the range of 4–20 nm (Supplementary Fig. S1b). In addition, based on Brunauer–Emmett–Teller (BET) calculation, the specific surface area of HPDA NPs was 157.141 m^2^·g^−1^.

The hollow structure greatly increased the specific surface area of HPDA NPs, which was very favorable for drug loading. Four different drugs—aspirin, ascorbic acid, tacrolimus, and simvastatin—were loaded on HPDA NPs, respectively. FTIR was used to verify whether the drugs were successfully loaded. The FTIR spectrum of aspirin@HPDA NPs showed that all other peaks corresponded to the peaks of aspirin, except for the specific peaks of HPDA NPs (Supplementary Fig. S2a). The other three, ascorbic acid@HPDA NPs, tacrolimus@HPDA NPs, and simvastatin@HPDA NPs, had similar results with aspirin@HPDA NPs (Supplementary Fig. S2b–d). These suggested that our HPDA NPs can successfully load these four drugs. The loading of drugs in HPDA NPs comes from two factors, one is the adhesion of drugs on the surface of PDA caused by the viscosity and the other is the *π*–*π* conjugate interaction between the ring structure in PDA and the ring structure in drug molecules. The above two factors can occur at the same time on the outer surface, the inner surface, and the cavity of the HPDA NPs; thus, the drugs can not only exist on the outer surface and the inner surface of the HPDA NPs but also exist in the cavity.

Next, we wanted to know the amount of each drug loaded in HPDA NPs. Ultraviolet (UV) absorption curve of each drug at different concentrations was measured (Supplementary Fig. S3a, d, g, j), which was further converted to the standard curve (Supplementary Fig. S3b, e, h, k). After each drug was loaded in HPDA NPs, the UV absorption value of the supernatant was measured and the drug concentration in the supernatant was obtained depending on corresponding drug standard curve (Supplementary Fig. S3c, f, i, l). Then, each drug loading rate and encapsulation rate was further calculated. Our results demonstrated that aspirin@HPDA NPs had 12.7% loading rate and 14.5% encapsulation rate, ascorbic acid@HPDA NPs had 30.8% loading rate and 44.5% encapsulation rate, tacrolimus@HPDA NPs had 16.3% loading rate and 19.5% encapsulation rate, and simvastatin@HPDA NPs had 10.7% loading rate and 12.0% encapsulation rate. Drug loading rate and encapsulation rate are influenced by many factors. The main reason is the structure of drug molecules. The strength of *π*–*π* conjugate interaction between drug molecules and polydopamine leads to the difference in drug loading capacity.^32^

Further, it was very important to evaluate if the drug loaded in HPDA NPs could be sustainably released from HPDA NPs efficiently. In general, our results demonstrated that all the drugs had similar release pattern. Supplementary Fig. S4 showed that aspirin@HPDA NPs and tacrolimus@HPDA NPs were more similar; they released about 50% of the loaded drugs within 4 days, then slowly released till 16 days and entered into the plateau after 16 days till day 30. On day 30, aspirin@HPDA NPs released about 90% of aspirin, whereas tacrolimus@HPDA NPs released about 79% of tacrolimus (Supplementary Fig. S4a, c and Supplementary Table S1). On the other hand, ascorbic acid@HPDA NPs and simvastatin@HPDA NPs were more similar; they released more than half the drugs at a fast rate within 4 days, then slowly released till 10 days and entered into the plateau after 10 days till day 30. On day 30, ascorbic acid@HPDA NPs and simvastatin@HPDA NPs released 90% and 87% of the loaded drug, respectively (Supplementary Fig. S4b, d and Supplementary Table S1). In each group, the rate of drug release was high during the first day of the experiment. This is because a small number of drugs adsorbed on the surface of NPs exist in amorphous form and are prone to brust release. These drug release data suggest that our HPDA NPs based drug sustained release system indeed can release loaded drugs sustainably till 30 tested days in the simulate body fluid. In addition to viscosity and *π*–*π* conjugate interaction, the hydrophilicity of the drug was the main factor affecting the release rate. More hydrophilic drugs had higher release rates and a higher release ratio at 30 days (Supplementary Fig. S4 and Supplementary Table S1). The slow release of drugs came from the structural disintegration of HPDA NPs. Also in the simulated body fluid, the structure of HPDA NPs was partially destroyed at 15 days compared to the hollow structure at 0 days (Supplementary Fig. S5a, b) and the structure disintegrated more severely at 30 days, almost completely damaged (Supplementary Fig. S5c).

To use HPDA NPs-based drug sustainable release system in vivo, especially in potential clinical application, we needed to know whether there was any cytotoxicity about this system. Cell Counting Kit-8 (CCK-8) assays were performed using rat BMSCs (rBMSCs). Basically, data from CCK-8 assays demonstrated that drugs loaded in the HPDA NPs did not affect cell viability. Because of the lower toxicity of the zinc ions, the HPDA NPs could slightly influence cell viability with the increase of HPDA NPs concentration (Supplementary Fig. S6). The influence of cell viability, however, was not significantly different when the HPDA NPs concentration was lower than 100 µg/mL compared to control cells without HPDA NPs (Supplementary Fig. S6). In our drug sustainable release system, the major component was polydopamine, which has high biocompatibility and low cytotoxicity. Data showed that cell viability was still more than 85% even at 200 µg/mL and HPDA NPs only had little impact on cell survival (Supplementary Fig. S6a). Seven-day CCK-8 experiments were also conducted. The results showed that the effect of the toxicity of nanomaterials on the relative cell viability decreased after the experiment time was prolonged and there was no significant difference in relative cell viability between the groups at low concentrations. However, the relative cell viability in the high concentration groups were significantly improved, which was because the drugs released by each drug sustained release system significantly improved the proliferation ability of cells (Supplementary Fig. S7).

**Fig. 3:**
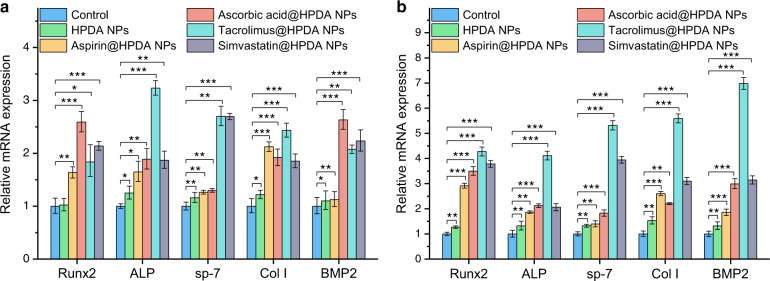
Effects of osteogenic drugs released from HPDA NPs on osteoblastic gene expressions of rBMSCs on day 7 (**a**) and 14 (**b**)

Next, it was important to know whether osteogenic drugs released from HPDA NPs could play corresponding roles in the osteoblast differentiation in vitro. The rBMSCs were cultured with HPDA NPs, aspirin@HPDA NPs, ascorbic acid@HPDA NPs, tacrolimus@HPDA NPs, or simvastatin@HPDA NPs and without any treatment as control. Then, quantitative real-time PCR (qRT-PCR) assays were used to evaluate five osteogenic gene expression, runt-related transcription factor 2 (*Runx2*), alkaline phosphatase (*ALP*), osterix (*sp-7*), *Col I*, bone morphogenetic protein 2 (*BMP2*), on days 7 and 14. These genes play important roles in osteogenic differentiation and can directly reflect the level of osteogenic differentiation.^42,43^ For example, *Runx2* is associated with the content of basic transcription factors in osteogenic differentiation. *Col I* is associated with the formation of extracellular matrix. *BMP2* is associated with the expression of bone matrix proteins and bone growth. Basically, data from gene expressions of these five genes showed that all four drugs released from HPDA NPs exhibited certain degree functions. As shown in Fig. 3a, b, HPDA NPs could slightly promote these five gene expressions compared to the control group on days 7 and 14. All four drugs could strongly enhance gene expressions of *Runx2*, *ALP*, and *Col I* compared to the control group on days 7 and 14. Drugs released from tacrolimus@HPDA NPs had stronger effects than that of aspirin, ascorbic acid, and simvastatin compared to the control group on day 14. In general, all four drugs had stronger effects on those five genes on day 14 compared to day 7 (Fig. 3a, b). These results clearly indicate that drugs released from HPDA NPs retain their functions.

**Fig. 4:**
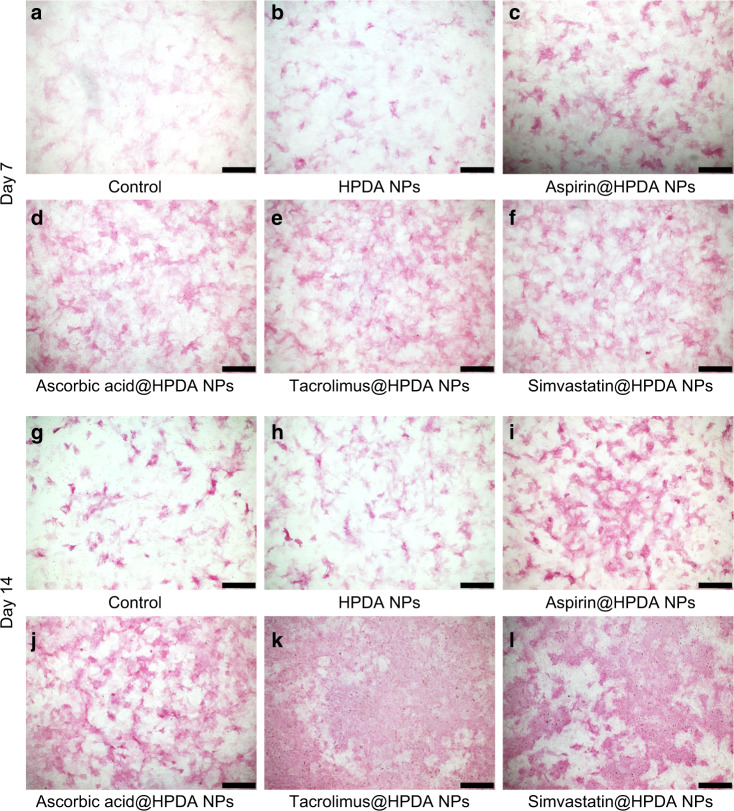
ALP staining of rBMSCs on day 7 (**a**–**f**) and 14 (**g**–**l**). **a**, **g** Control. **b**, **h** HPDA NPs. **c**, **i** Aspirin@HPDA NPs. **d**, **j** Ascorbic acid@HPDA NPs. **e**, **k** Tacrolimus@HPDA NPs. **f**, **l** Simvastatin@HPDA NPs. Scale bar is 500 μm

**Fig. 5:**
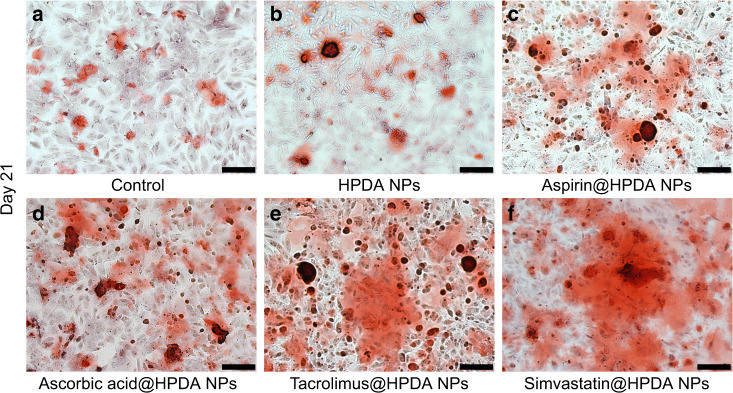
Alizarin red S staining of rBMSCs on day 21. **a** Control. **b** HPDA NPs. **c** Aspirin@HPDA NPs. **d** Ascorbic acid@HPDA NPs. **e** Tacrolimus@HPDA NPs. **f** Simvastatin@HPDA NPs. Scale bar is 200 μm

To further verify qRT-PCR results, direct ALP staining and alizarin red S staining assays were performed. Figure 4a–l clearly demonstrated that all four drugs released from aspirin@HPDA NPs, ascorbic acid@HPDA NPs, tacrolimus@HPDA NPs, or simvastatin@HPDA NPs dramatically promoted ALP activity increase and rBMSCs proliferation on days 7 and 14 compared to the control group and HPDA NPs-treated group, especially on day 14. In addition to the staining photos, the quantitative analysis of ALP more intuitively showed the difference in ALP activity among the groups, which proved the effective effect of the drug sustainable release system (Supplementary Fig. S8). Data from alizarin red S staining also showed that all four drugs released from aspirin@HPDA NPs, ascorbic acid@HPDA NPs, tacrolimus@HPDA NPs, or simvastatin@HPDA NPs significantly increased calcium deposits or mineralization level on day 21 compared to the control group and HPDA NPs-treated group (Fig. 5a–f). Both stainings also showed that tacrolimus and simvastatin had stronger effects on osteogenic differentiation compared to aspirin and ascorbic acid (Figs. 4 and 5). All these results were consistent with the results of qRT-PCR. These data further suggest that drugs released from the HPDA NPs retain effective functions, osteogenic differentiation and proliferation in vitro, and have sustainable release property.

**Fig. 6:**
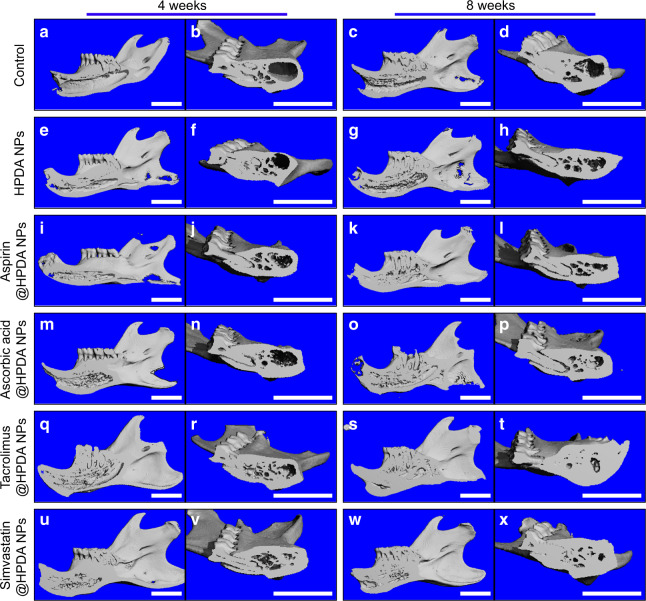
In vivo Micro-CT images of rat mandible after 4 and 8 weeks post surgery. **a**, **b** Control, 4 weeks. **c**, **d** Control, 8 weeks. **e**, **f** HPDA NPs, 4 weeks. **g**, **h** HPDA NPs, 8 weeks. **i**, **j** Aspirin@HPDA NPs, 4 weeks. **k**, **l** Aspirin@HPDA NPs, 8 weeks. **m**, **n** Ascorbic acid@HPDA NPs, 4 weeks. **o**, **p** Ascorbic acid@HPDA NPs, 8 weeks. **q**, **r** Tacrolimus@HPDA NPs, 4 weeks. **s**, **t** Tacrolimus@HPDA NPs, 8 weeks. **u**, **v** Simvastatin@HPDA NPs, 4 weeks. **w**, **x** Simvastatin@HPDA NPs, 8 weeks. Scale bar is 5 mm

**Fig. 7:**
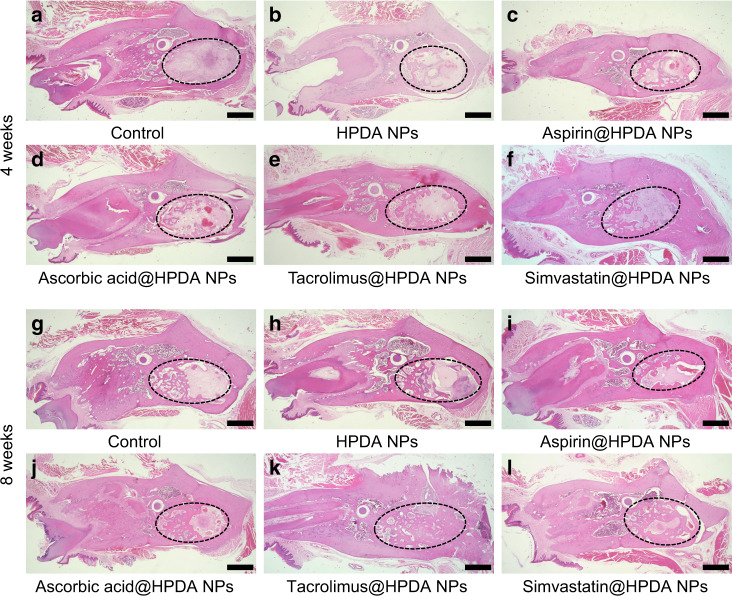
H&E staining of the rat mandibles after 4 weeks (**a**–**f**) and 8 weeks (**g**–**l**) post surgery. **a**, **g** Control. **b**, **h** HPDA NPs. **c**, **i** Aspirin@HPDA NPs. **d**, **j** Ascorbic acid@HPDA NPs. **e**, **k** Tacrolimus@HPDA NPs. **f**, **l** Simvastatin@HPDA NPs. Scale bar is 1 mm

Importantly, to know whether drug sustainable release system could effectively release drug in the HPDA NPs in vivo, therefore, the in vivo degradation performance was studied first. HPDA NPs were injected subcutaneously into rats. Four weeks later, the skin was cut open and the black nanomaterials that remained under the skin were collected and dissolved, and were observed by TEM. TEM images showed that HPDA NPs were degraded in vivo and their structure was destroyed, which allows the effectively drug release in the body (Supplementary Fig. S9). Then, animal experiments were performed using rat tooth extraction fossa defect model to evaluate effects of HPDA NPs loaded with different drugs on bone repair after 4 and 8 weeks post implant. Images from micro-computed tomography (Micro-CT) showed that only a very small amount of new bone was found in the cavity of the tooth extraction fossa in the control group with obvious large defect bone volume on week 4 (Fig. 6a, b). After 8 weeks, new bone mass slightly increased to reach 30.5% of the total defect volume (Fig. 6c, d and Supplementary Fig. S10). Compared to the control group, Micro-CT images from the HPDA NPs group showed that the new bone formation was slightly higher (Fig. 6e–h). The volume of new bone fraction in the tooth extraction fossa was 37.1% at 4 weeks and increased to 46.2% at 8 weeks (Supplementary Fig. S10). By comparison, all four osteogenic drugs released from HPDA NPs had significant effects on the new bone formation on weeks 4 and 8 (Fig. 6i–x and Supplementary Fig. S10). Although there were cavities in the tooth extraction fossa in aspirin@HPDA NPs and ascorbic acid@HPDA NPs groups at 4 weeks, the volume of cavities was smaller and there were low-density images in the cavities, indicating that there were not only mature new bones but also immature and loose bone tissues (Fig. 6i, j, m, n). With time increase, mature bone tissues and new bone volume from these two groups significantly increased, compared to control and HPDA NPs groups at 8 weeks (Fig. 6k, l, o, p). Interestingly, osteogenic drugs released from tacrolimus@HPDA NPs and simvastatin@HPDA NPs were consistent with in vitro data, which had much stronger osteogenesis promotion in vivo compared to aspirin and ascorbic acid on weeks 4 and 8 (Fig. 6q–x), especially regenerated new bones could almost cover the bone defect area at 8 weeks (Fig. 6s, t, w, x). Quantitative data showed that the volume of new bones had exceeded 90% of the total defect volume and statistical analysis showed that tacrolimus and simvastatin had stronger effects on bone regeneration compared to aspirin and ascorbic acid (Supplementary Fig. S10). In addition to Micro-CT data, images of hematoxylin and eosin (H&E) staining from the same tissues further verified what we saw from the images of Micro-CT (Fig. 7). These data indicate that the HPDA NPs itself has weaker effects on the new bone formation in vivo. All four osteogenic drugs released from the HPDA NPs retain their functions to effectively promote new formation and the effects can last 8 weeks at least in vivo, which suggests that the drug loaded in the HPDA NPs can sustainably release in vivo. Among the four osteogenic drugs, tacrolimus has the strongest effect on the bone regeneration.

Lastly, it was important to know whether there was any toxicity of HPDA NPs in vivo. Therefore, functions of the liver and kidney were evaluated. Each index of liver function and kidney function did not show any significant difference compared to control group at 8 weeks (Supplementary Fig. S11). Further, data from H&E staining also did not indicated that there was any pathological change in the heart, liver, spleen, lung, and kidney at 8 weeks (Supplementary Fig. S12). These data together indicate that our HPDA NPs-based drug sustained system is safe and has potential in the clinical application.

## Discussion

In the clinic, bone defect seriously affects the physical and mental health of patients.^44^ Allogeneic bone transplantation is faced with the limitation of donor sources and immune rejection.^45^ Autologous bone transplantation is faced with self-damage.^46^ Therefore, the use of osteogenic drugs is ideal to induce bone tissue regeneration. The osteogenic drug metabolism, however, is rapidly resulting in low efficacy when the topical administration is used, which also cause more side effects because of requiring repeatable administration. It is necessary to develop local implantable sustained release system that can deliver drugs over a long period of time. In this study, we created a safe and low-toxic HPDA NPs-based drug delivery system that could efficiently carry the osteogenic drugs and slowly release osteogenic drugs in vitro and in vivo, leading to enhanced osteoblast differentiation in vitro and effective new bone regeneration at the bone defect area in vivo.

Under the dual action of the viscosity of polydopamine and *π*–*π* conjugation between polydopamine and drug molecules, the drug loading capacity was greatly increased. With the improvement of drug loading capacity, fewer carriers can load more drugs, which can effectively reduce the dosage of carriers and greatly benefit the improvement of safety. The gradual degradation of nanomaterials in the physiological environment allows drugs to be gradually released, to play a bone-promoting role. In our study, the drug can be released continuously for as long as a month and the effect lasts for a long time (Supplementary Fig. S4 and Supplementary Table S1), and the effect is more significant. Cell experiment results from the rBMSCs showed that the osteogenic drugs released from the HPDA NPs could effectively promote the expression of osteogenic genes, enhance the activity of ALP, and promote the formation of calcium nodules in vitro (Figs. 3–5). These indicate that the drugs released from the HPDA NPs retain the biological functions and effectively promote osteoblast differentiation and proliferation in vitro. Aspirin inhibits the formation of inflammatory cells and the osteoclast differentiation of rBMSCs.^36^ Ascorbic acid inhibits the formation of osteoclasts and promotes the formation of mineralization centers.^37^ Tacrolimus induces differentiation and proliferation of rBMSCs into osteoblasts.^38^ Simvastatin stimulates bone morphogenetic protein and promotes the production of Col I.^39^ Notably, the non-drug-loaded HPDA NPs also showed a lower level of osteogenic ability. Both the viscosity and the rough structure in mesoporous scale of HPDA NPs are conducive to the adhesion and proliferation of osteoblasts, thus improving the effect of osteogenesis.

Importantly, these four osteogenic drugs could be efficiently released into the bone defect area of tooth extraction fossa locally and retained the biological effects for a long time leading to new bone regeneration at the bone defect area in vivo (Figs. 6 and 7). Compared to the control group, the bone regeneration rates were significantly higher in all four osteogenic drug-treated groups, especially the tacrolimus@HPDA NPs-treated group. Further, toxicity data demonstrated that there was no difference of functions of the liver and kidney, and no difference of morphology of the heart, liver, spleen, lung, and kidney compared to the control group (Supplementary Figs. S11 and S12). The polydopamine is a substance that is naturally present in the body and is extremely low in toxicity. The drug-loaded HPDA NPs were only applied locally and most drugs in HPDA NPs could be released locally at the bone defect area. Therefore, the efficacy of the drugs can be guaranteed to only use a very low dosage in order to achieve optimal effect with much less side effects. These in vivo data strongly indicate the safety of HPDA NPs-based drug sustainable release system. Our design can well solve the problem of bone loss caused by the absorption of the alveolar ridge after tooth extraction, can provide enough bone mass and good sclerotin for the later denture repair and implant implantation, and has great potential application in the clinical practice.

## Conclusions

In conclusion, the HPDA NPs created herein have lower toxicity and high biocompatibility in vitro and in vivo. The HPDA NPs can efficiently load different osteogenic drugs, sustainably release these drugs to effectively promote osteoblast differentiation and proliferation in vitro, and new bone formation to repair defect bone area in vivo. Our data indicate that the tacrolimus released from tacrolimus@HPDA NPs has the strongest effect on the new bone formation in vivo. Next, we plan to use tacrolimus@HPDA NPs to modify titanium implants to improve the treatment of large bone defects. In addition, we still cannot conclude which drug sustained release system is better due to their different mechanisms. It would be the best choice to clarify the mechanism of each drug sustained release system, integrate their functions, and promote the best bone regeneration performance through synergistic effect in the future.

## Materials and methods

### Preparation of ZIF-8

Here, 2-MIL (41.06 mg; MilliporeSigma, St. Louis, MO, USA) was dissolved in 10 mL of absolute methanol to form 2-MIL solution (50 mmol·L^−1^). Then, 74.37 mg of Zn(NO_3_)_2_·6H_2_O (MilliporeSigma) was dissolved in 10 mL of absolute methanol to form Zn(NO_3_)_2_ solution (25 mmol·L^−1^). These two solutions were mixed together at room temperature, kept stationary for 1 h, and spun at 7 000 r.p.m. for 10 min. The white precipitation was ZIF-8 after supernatant was removed. ZIF-8 precipitation was washed with absolute methanol (Sinopharm, Shanghai, China) for three times, then dispersed in 4 mL of absolute methanol for further use.

### Preparation of HPDA NPs

Dopamine hydrochloride (4.74 mg; MilliporeSigma) was dissolved in 5 mL of absolute methanol to form dopamine solution (5 mmol·L^−1^), then mixed with above ZIF-8 solution, heated at 60 °C for 10 h, and centrifuged at 7 000 r·min^−1^ for 10 min to get a black precipitation of HPDA NPs. The precipitation of HPDA NPs was washed with absolute methanol for three times and dispersed in 1 mL of absolute ethanol (Sinopharm) for further use.

### Characteristics of HPDA NPs

TEM (JEOL, Tokyo, Japan) was used to perform TEM analysis with an accelerating voltage of 200 kV. X-ray diffractometer (XRD, PANalytical B.V., Almelo, Netherlands) was used to perform XRD analysis with Cu K radiation (*λ* = 1.541 8 Å). FTIR spectrometer (Brucker, Karlsruhe, Germany) was used to perform FTIR analysis with KBr as the background. X-ray photoelectron spectrometer (XPS, VG, London, Britain) was used to perform XPS analysis with an Mg Kα excitation (1 253.6 eV). BET analyzer (NOVA 4200e, Quantachrome, Florida, USA) was used to perform N_2_ adsorption/desorption isotherm.

### Loading and releasing properties of HPDA NPs

Four drugs—aspirin, ascorbic acid, tacrolimus, and simvastatin (Solarbio, Beijing, China)—were dissolved in absolute ethanol at 2 mg·mL^−^^1^. In the process of drug loading, 2 mg·mL^−^^1^ HPDA NPs solution and drug solution were mixed in the same volume. Twenty-four hours later, the supernatant was collected by centrifugation at 7 000 r·min^−1^ for 10 min to obtain aspirin@HPDA NPs, ascorbic acid@HPDA NPs, tacrolimus@HPDA NPs, and simvastatin@HPDA NPs. FTIR was used to verify that the drugs have been successfully loaded. Then, they were washed by deionized water and resuspended in 1 mL water for further use. At the same time, the ultraviolet absorption spectrum of supernatant after drug loading was determined. The ultraviolet absorption value of the supernatant was substituted into the standard curve to obtain the drug concentration in the supernatant. The drugs in the supernatant are the drugs that are not loaded, so the amount of the loaded drugs can be obtained by combining the total amount of the drug. Computational formula: drug loading rate = mass of loaded drug/(mass of loaded drug + mass of HPDA NPs); drug encapsulation rate = mass of loaded drug/total mass of feeding drug. Next, each drug-loaded HPDA NPs was diluted in simulate body fluid (Jieshikang, Qingdao, China) to test the release at 0, 0.25, 0.5, 1, 2, 4, 6, 8, 10, 12, 16, 18, 20, 22, 24, 26, 28, and 30 days, respectively. Samples at different time points were centrifuged and the UV absorption spectra of the supernatant were measured. The release percentages were calculated by the UV absorption values of the supernatant depending on the standard curve.

### Cell cultures

The rBMSCs were collected from rat femur, washed with phosphate-buffered saline (PBS) containing 500 μg·mL^−^^1^ streptomycin and 500 U·mL^−^^1^ penicillin (Hyclone, Beijing, China), cultured with low-glucose Dulbecco’s modified Eagle medium (DMEM; Solarbio), 15% fetal bovine serum (Bioind, Guangzhou, China), 100 U·mL^−^^1^ of penicillin G, and 100 μg·mL^−^^1^ of streptomycin, and incubated at 37 °C in a humidified 5% CO_2_ atmosphere. The medium was replaced every 3 days and sub-cultured after 80% confluence. Cells from a third passage were used for experiments.

### Cytotoxicity assay

CCK-8 (Beyotime, Shanghai, China) staining was used to evaluate cytotoxicity of HPDA NPs on rBMSCs. This experiment was divided into five groups as follows: HPDA NPs group, aspirin@HPDA NPs group, ascorbic acid@HPDA NPs group, tacrolimus@HPDA NPs group, and simvastatin@HPDA NPs group. The rBMSCs were cultured at 4000 cells per well in 96-well plate. After 24 h, different concentrations of each drug-loaded HPDA NPs, 0, 25, 50, 100, 150, and 200 μg·mL^−^^1^ were added into corresponding wells and continued to culture for 24 h or 7 days. Then, medium was discarded carefully and 20 μL CCK-8 reagent in 180 μL DMEM was added into each well, incubated for 1–2 h at 37 °C, and measured the optical density (OD) values at 450 nm (UV absorption spectrophotometer, UV2600, Shimadzu, Tokyo, Japan). Each experiment was repeated five times.

### Quantitative real-time PCR assay

To evaluate whether the treated cells were differentiated to osteoblasts in our studying conditions, osteogenic-related genes—*Runx2*, *ALP*, *sp-7*, *Col I*, and *BMP2*—were measured. The rBMSCs were cultured at 100 000 cells per well in 6-well plates and treated by HPDA NPs, aspirin@HPDA NPs, ascorbic acid@HPDA NPs, tacrolimus@HPDA NPs, and simvastatin@HPDA NPs at 100 μg·mL^−^^1^, respectively. All the rBMSCs in each group were harvested on days 7 and 14, then total RNA was extracted by Trizol (Takara, Dalian, China), reverse-transcribed using cDNA synthesis kit (Takara), and run quantitative PCR assays with PCR mastercycler (Mx3005P, Agilent, Palo Alto, USA) using 5× master mix (Takara). All primers used in this study were obtained from Takara and primer sequences were shown in Supplementary Table S2. Relative gene expression levels were normalized with the *β-actin*.

### ALP staining and ALP activity assay

To further evaluate osteogenic effects of drug HPDA NPs sustainable release systems, ALP staining, an early marker of osteoblast differentiation, was performed. The rBMSCs were cultured at 30 000 cells per well in 24-well plates and treated by HPDA NPs, aspirin@HPDA NPs, ascorbic acid@HPDA NPs, tacrolimus@HPDA NPs, and simvastatin@HPDA NPs at 100 μg·mL^−^^1^, respectively. The rBMSCs on days 7 and 14 were fixed by stationary liquid and stained using ALP staining kit (MilliporeSigma). Images were photographed under an optical microscope. Further, ALP activity was quantitatively measured using ALP activity colorimetric assay kit (Beyotime). The ALP activity was calculated depending on the OD value at 405 nm. Data were expressed as μmol pNPP/min/mg protein.

### Alizarin red S staining

Alizarin red S staining, another marker of osteoblast differentiation, was used to evaluate the mineralization of rBMSCs. The rBMSCs were cultured at 50 000 cells per well in 12-well plates and treated by HPDA NPs, aspirin@HPDA NPs, ascorbic acid@HPDA NPs, tacrolimus@HPDA NPs, and simvastatin@HPDA NPs at 100 μg·mL^−^^1^, respectively. After culturing for 21 days, rBMSCs were washed three times with 1× PBS, fixed with 95% ethanol for 10 min, washed three times with deionized water, stained with 1% alizarin red S solution (Solarbio) for 30 min, rinsed with deionized water, and observed under an optical microscope (IX71, Olympus, Tokyo, Japan).

### In vivo animal experiments

All our animal experiments were performed in accordance with the Guidelines for Care and Use of Laboratory Animals of Jilin University and approved by the Animal Ethics Committee of Jilin University. Sixty male Sprague–Dawley rats (~180 g body weight) were used in this study. Rats were divided into six groups randomly as follows: control group, HPDA NPs group, aspirin@HPDA NPs group, ascorbic acid@HPDA NPs group, tacrolimus@HPDA NPs group, and simvastatin@HPDA NPs group. On days −9, −6, and −3 before tooth extraction, right incisor was cut off along the gingiva with a turbo handpiece. On day 0, rats were anesthetized with isoflurane and the gingival was separated using gingival separator. The right mandibular central incisor was clamped with a needle holder and extracted the whole tooth along the curve direction of the root. Then, each kind of drug-loaded HPDA NPs for each group was put into the tooth extraction fossa and sutured gums directly with 3–0 absorbable suture. In each group, the dosage of the drug sustained release system was consistent, with 100 μg for HPDA NPs, aspirin@HPDA NPs, ascorbic acid@HPDA NPs, tacrolimus@HPDA NPs, and simvastatin@HPDA NPs. The control group did not have any treatment. Five rats from each group were killed after 4 or 8 weeks post treatment and the mandible from each mouse was dissected for further micro-CT (μCT50, Scanco Medical AG, Zurich, Switzerland) analyses to measure the shape and volume of new bone. The same tissues were also used to perform H&E staining.

In addition, blood sample from each rat was collected for liver and kidney function analyses, and the heart, liver, spleen, lung, and kidney were also collected for pathological analyses by H&E staining, to evaluate the biosafety of different drug sustained release systems in vivo.

### Statistical analysis

All experiments were repeated three times or more. All the data were presented as mean ± SD. Then, statistical analysis was performed for the evaluation of statistical significances among the groups using Statistical Package for Social Sciences software. *P* < 0.05 was considered to be statistical significant.

## Supplementary information

SUPPLEMENTAL MATERIAL
