# Supplementary material for: Construction of hollow polydopamine nanoparticle based drug sustainable release system and its application in bone regeneration
Source: Int J Oral Sci. 2021 Aug 18;13:27. doi: 10.1038/s41368-021-00132-6 (PMC8373924; doi:10.1038/s41368-021-00132-6)

**Supplementary**

Construction of hollow polydopamine nanoparticle based drug sustainable release system and its application in bone regeneration

Lu Wang,^1,2^ Shuwei Liu,^3^ Chunxia Ren,^1,2^ Siyuan Xiang,^3^ Daowei Li,^2^ Xinqing Hao,^1,2^ Shilei Ni,^2^ Yixin Chen,^3^ Kai Zhang,^3,*^ and Hongchen Sun^1,2,*^

^1^Department of Oral Pathology, Hospital of Stomatology, Jilin University, Changchun, 130021, China

^2^Jilin Provincial Key Laboratory of Tooth Development and Bone Remodeling, School and Hospital of Stomatology, Jilin University, Changchun, 130021, China

^3^State Key Laboratory of Supramolecular Structure and Materials, College of Chemistry, Jilin University, Changchun, 130012, China

*Correspondence to:

Dr. Hongchen Sun

Department of Oral Pathology, Hospital of Stomatology, Jilin University, Changchun, 130021, China

Email: [hcsun@jlu.edu.cn](mailto:hcsun@jlu.edu.cn)

Tel: +86-24-31927800; Fax: +86-24-31927811

Dr. Kai Zhang

State Key Laboratory of Supramolecular Structure and Materials, College of Chemistry, Jilin University, Changchun, 130012, China

Email: [zk@jlu.edu.cn](mailto:zk@jlu.edu.cn)

Tel: +86-431-85168283


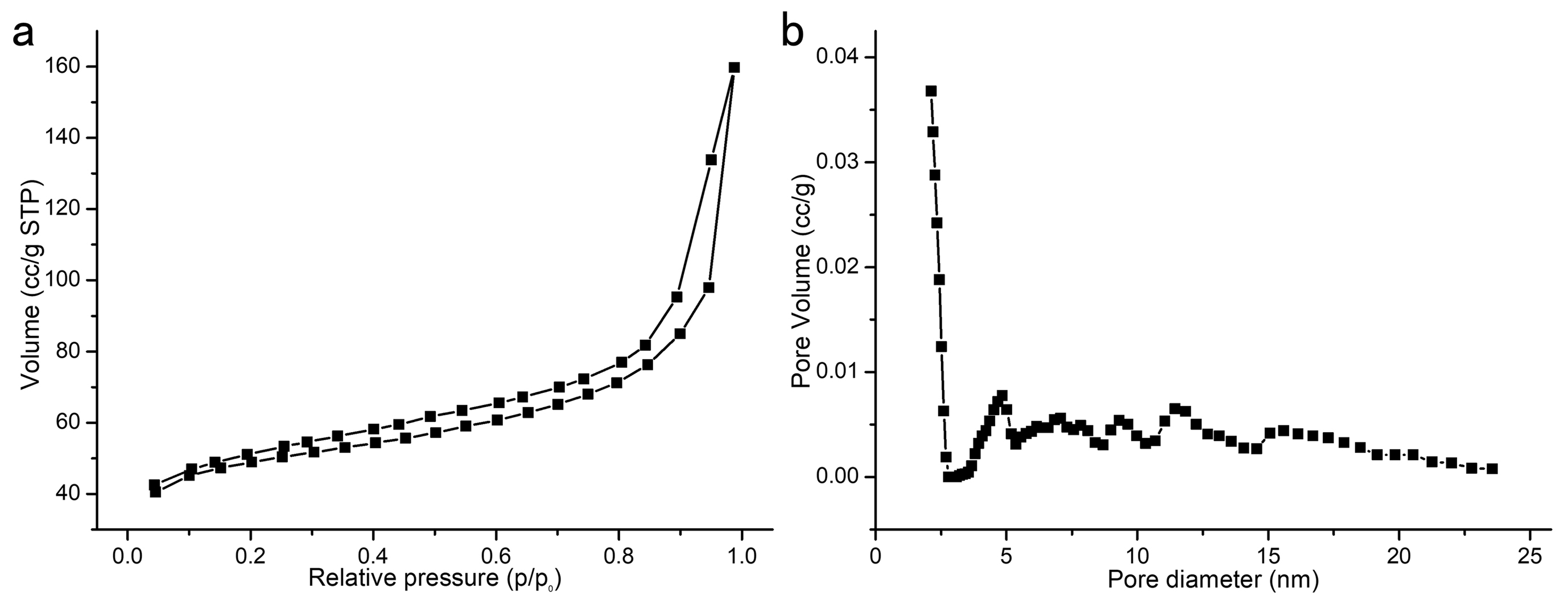


**Figure S1.** (a) N_2_ adsorption/desorption isotherms and (b) corresponding pore size distribution of HPDA NPs.


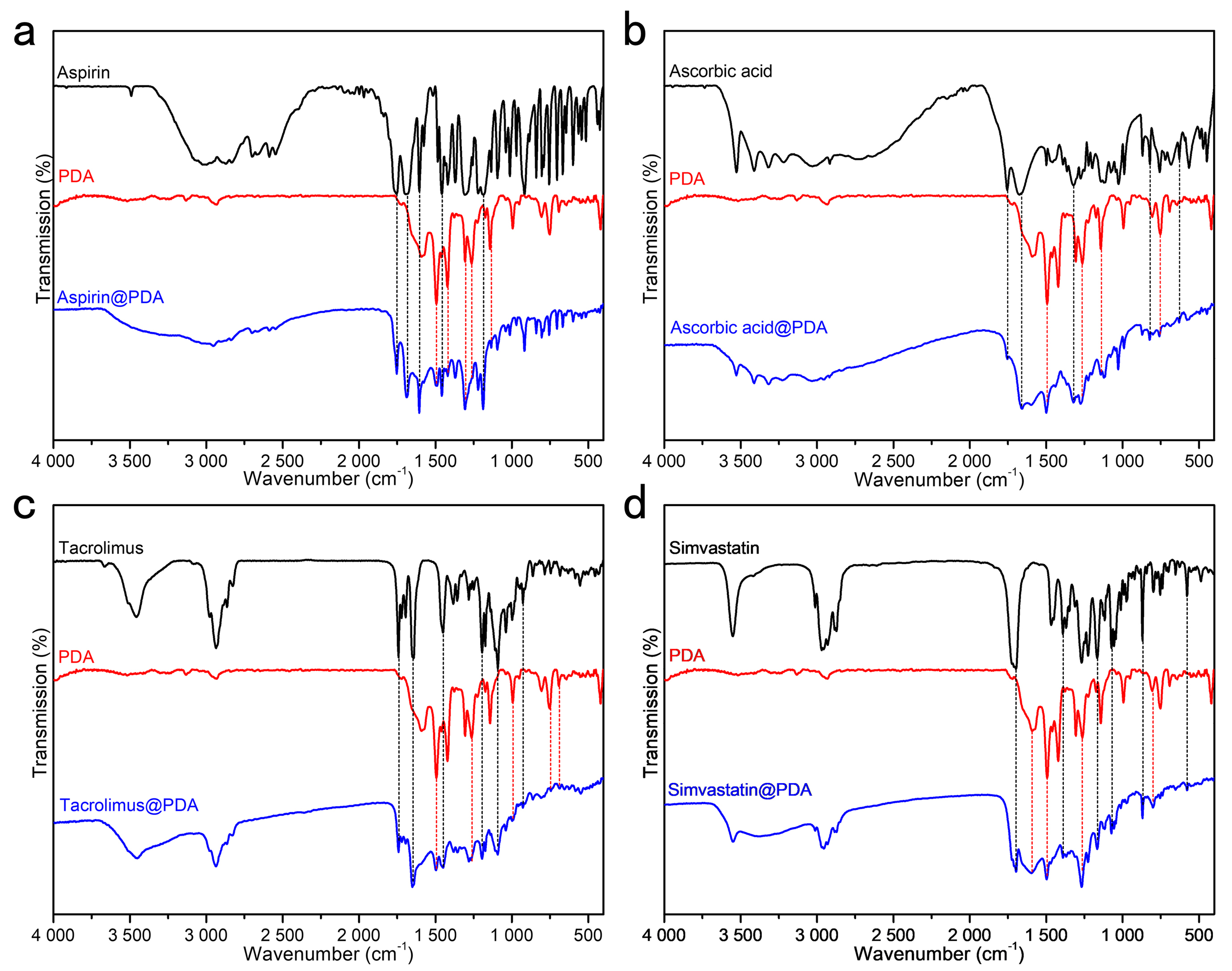


**Figure S2.** FTIR spectrum of each drug loaded in the HPDA NPs compared to each drug only and HPDA NPs only. (a) Aspirin@HPDA NPs. (b) Ascorbic acid@HPDA NPs. (c) Tacrolimus@HPDA NPs. (d) Simvastatin@HPDA NPs. It is worth noting that the FTIR spectrum of HPDA NPs is reused for a more intuitive comparison.


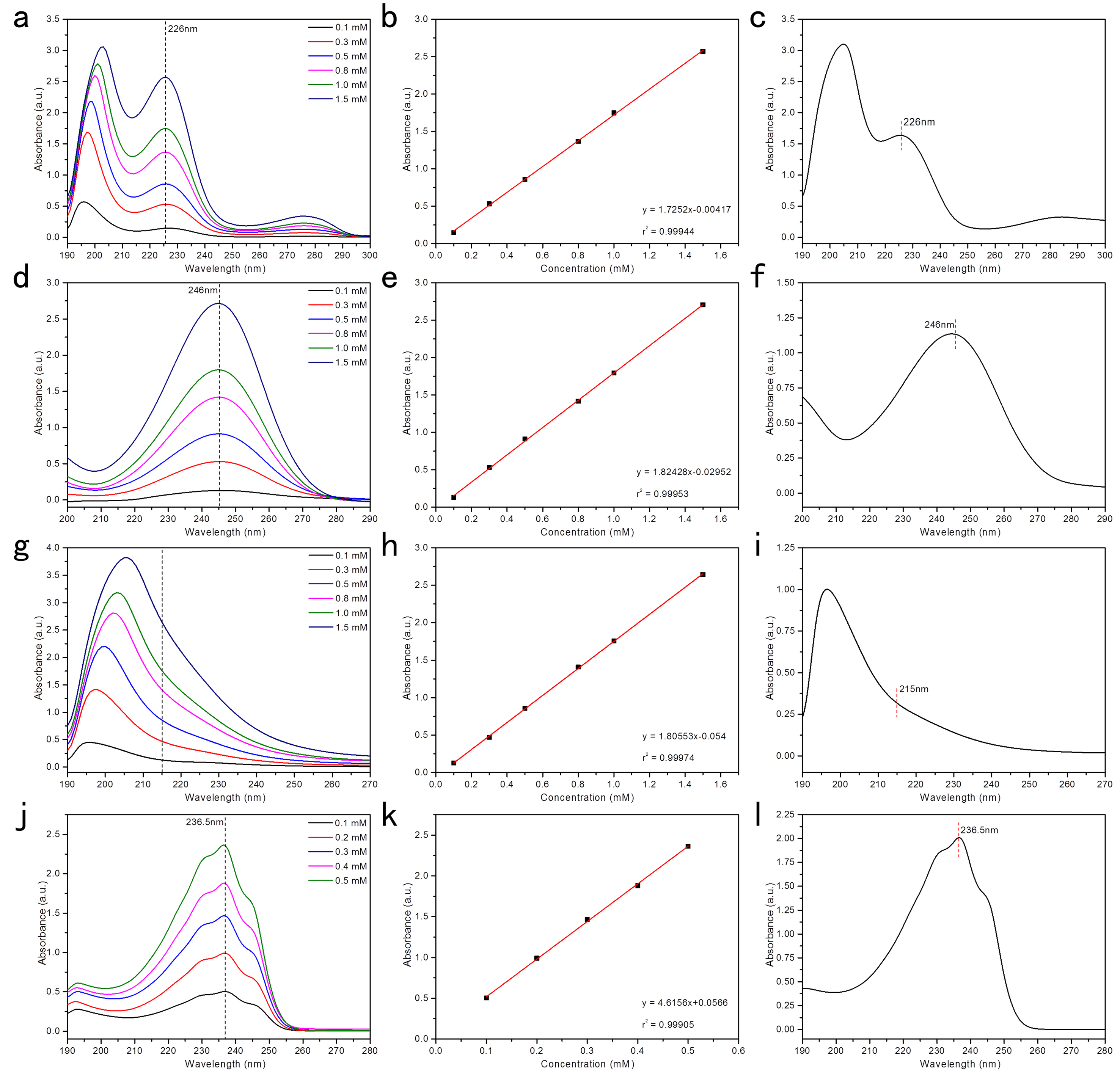


**Figure S3.** Measurements of loading rate and encapsulation rate of each drug in the HPDA NPs by the standard curve method. (a) The UV absorption spectra of aspirin. (b) Standard curve of aspirin. (c) Loading rate and encapsulation rate of aspirin, 12.7 % and 14.5 %, respectively. (d) The UV absorption spectra of ascorbic acid. (e) Standard curve of aspirin. (f) Loading rate and encapsulation rate of ascorbic acid, 30.8 % and 44.5 %, respectively. (g) The UV absorption spectra of tacrolimus. (h) Standard curve of aspirin. (i) Loading rate and encapsulation rate of tacrolimus, 16.3 % and 19.5 %, respectively. (j) The UV absorption spectra of simvastatin. (k) Standard curve of aspirin. (l) Loading rate and encapsulation rate of simvastatin, 10.7 % and 12.0 %, respectively.


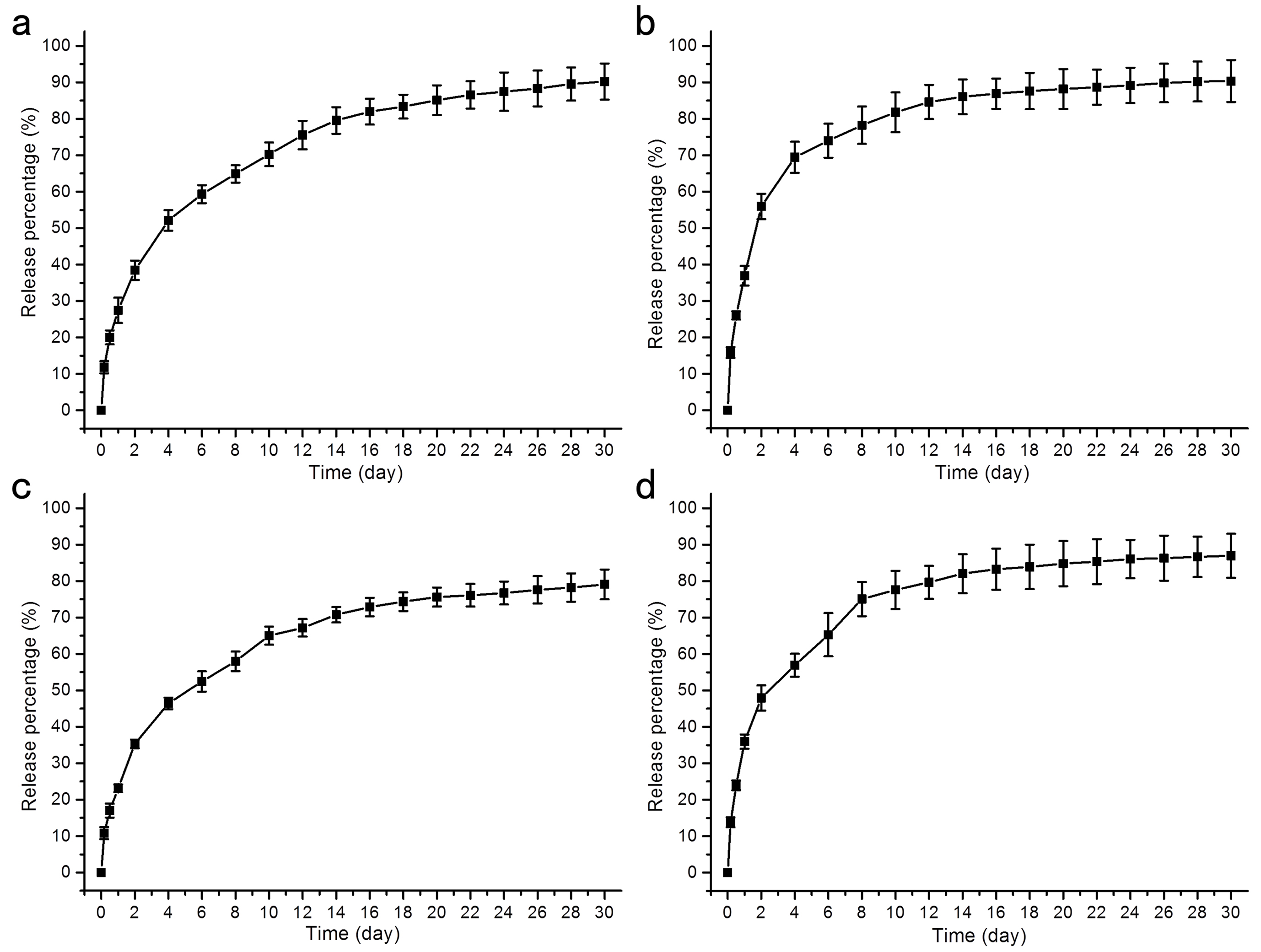


**Figure S4.** Drug release profiles from HPDA NPs in simulated body fluids. (a) Aspirin@HPDA NPs. (b) Ascorbic acid@HPDA NPs. (c) Tacrolimus@HPDA NPs. (d) Simvastatin@HPDA NPs.

**Table S1.** Drug release percentage from HPDA NPs at different time points in the simulated body fluids


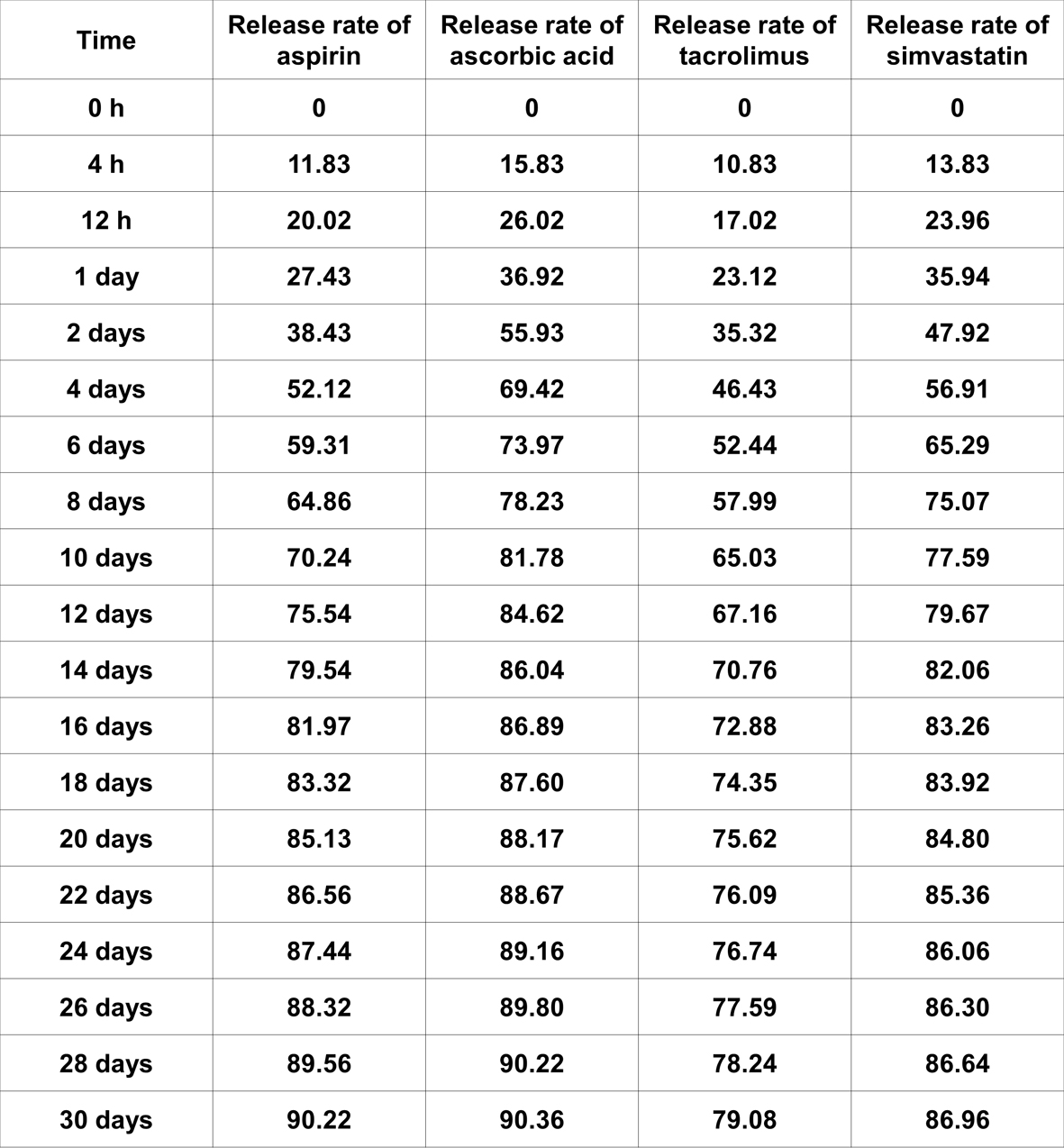


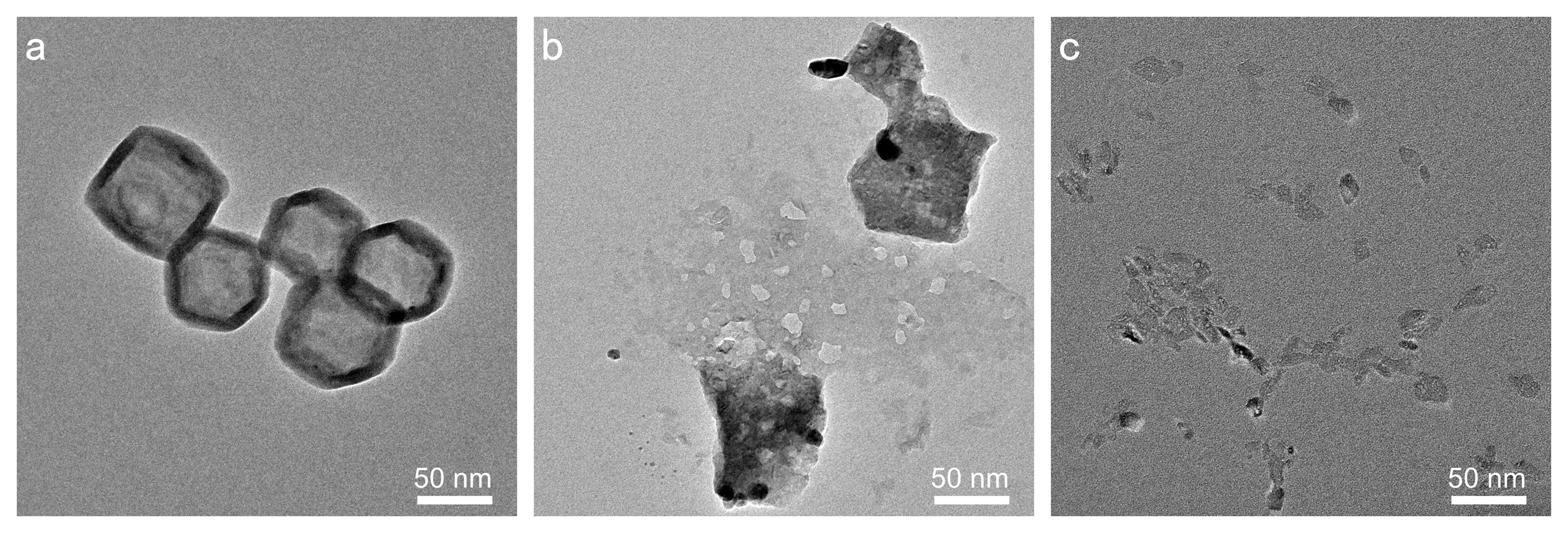


**Figure S5.** TEM images of HPDA NPs co-cultured with simulated body fluids after 0 (a), 15 (b) and 30 days (c).


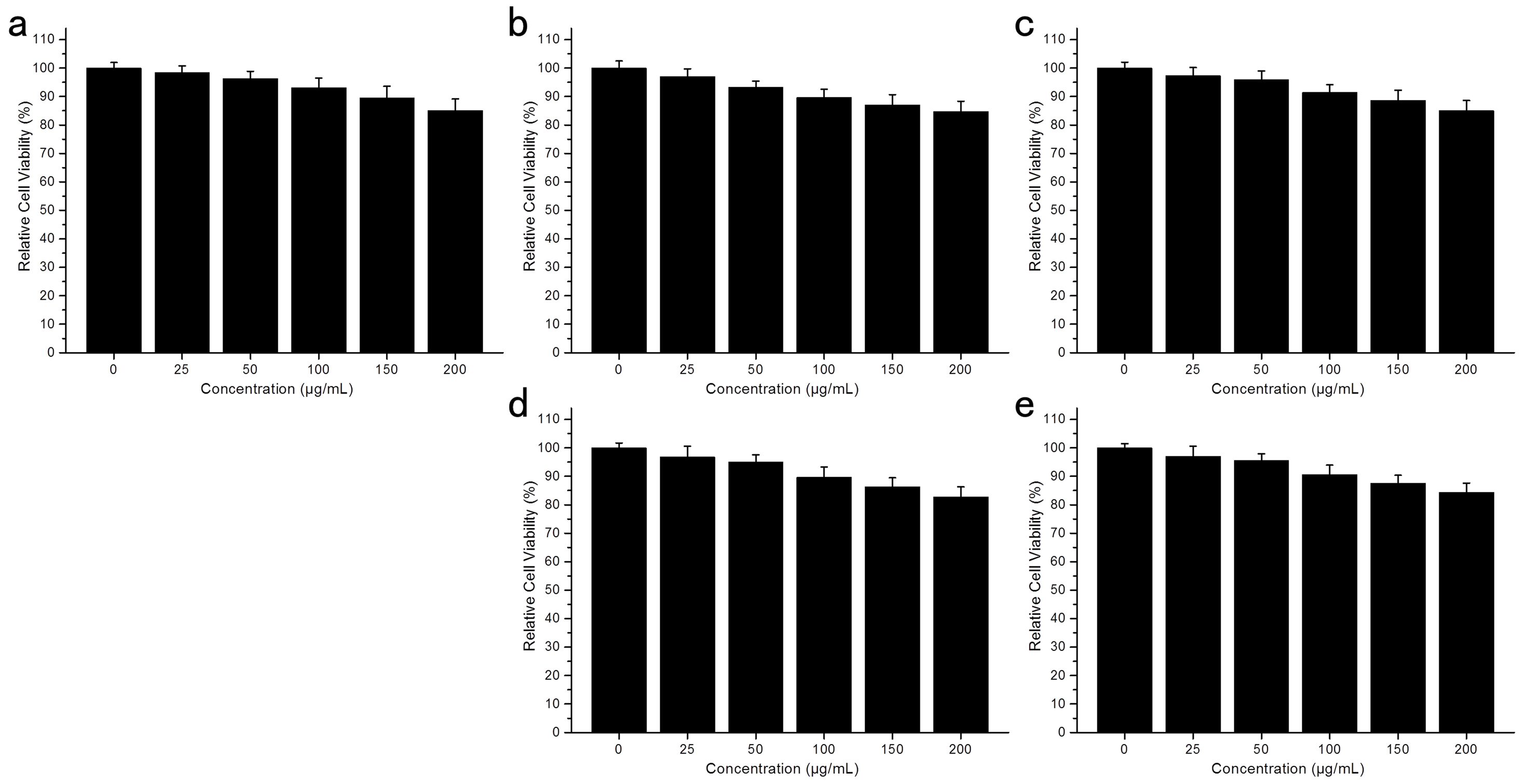


**Figure S6.** CCK-8 assays to evaluate the cytotoxicity of the NPs at 24 h. (a) HPDA NPs. (b) Aspirin@HPDA NPs. (c) Ascorbic acid@HPDA NPs. (d) Tacrolimus@HPDA NPs. (e) Simvastatin@HPDA NPs.


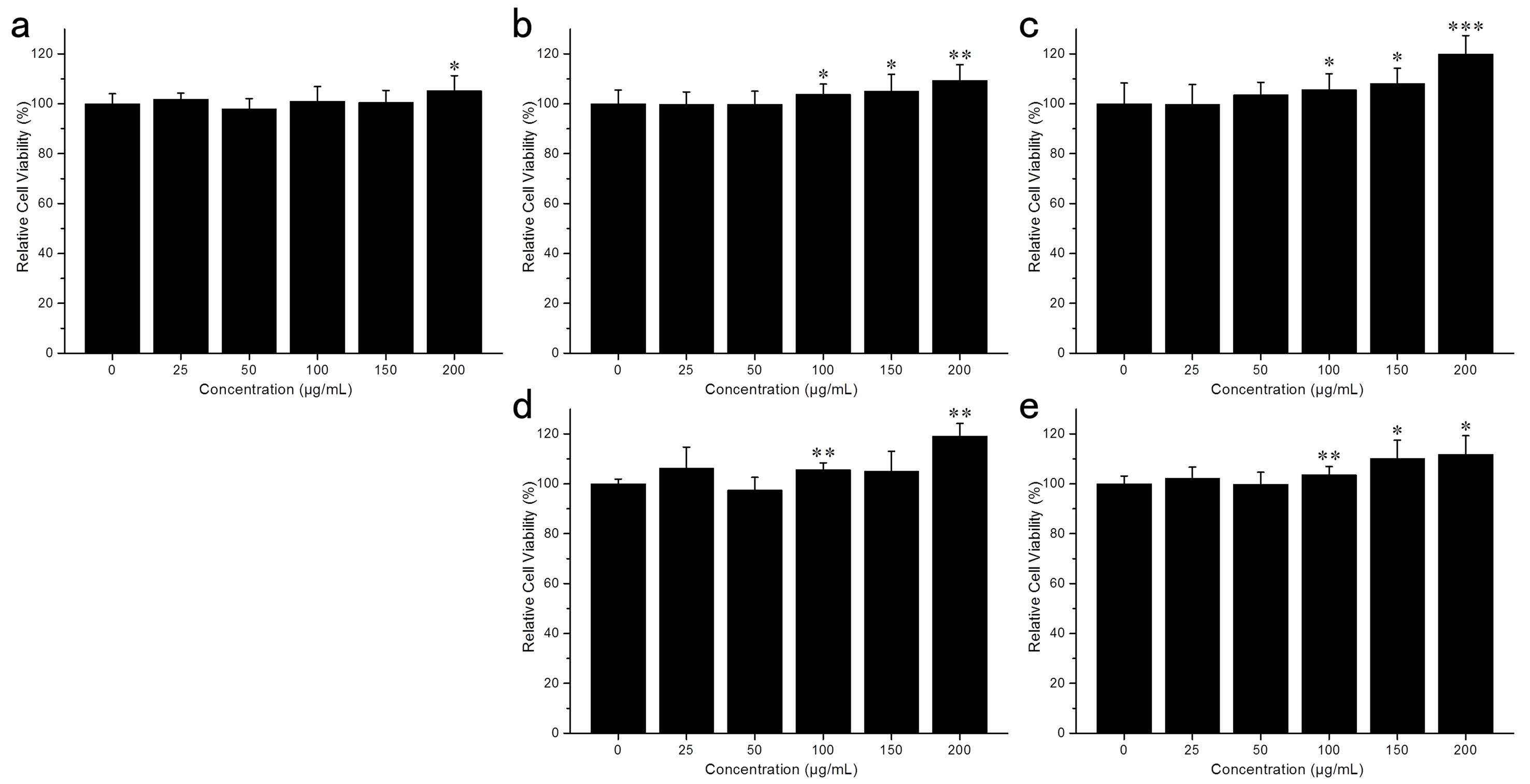


**Figure S7.** CCK-8 assays to evaluate the cytotoxicity of the NPs at 7 days. (a) HPDA NPs. (b) Aspirin@HPDA NPs. (c) Ascorbic acid@HPDA NPs. (d) Tacrolimus@HPDA NPs. (e) Simvastatin@HPDA NPs.


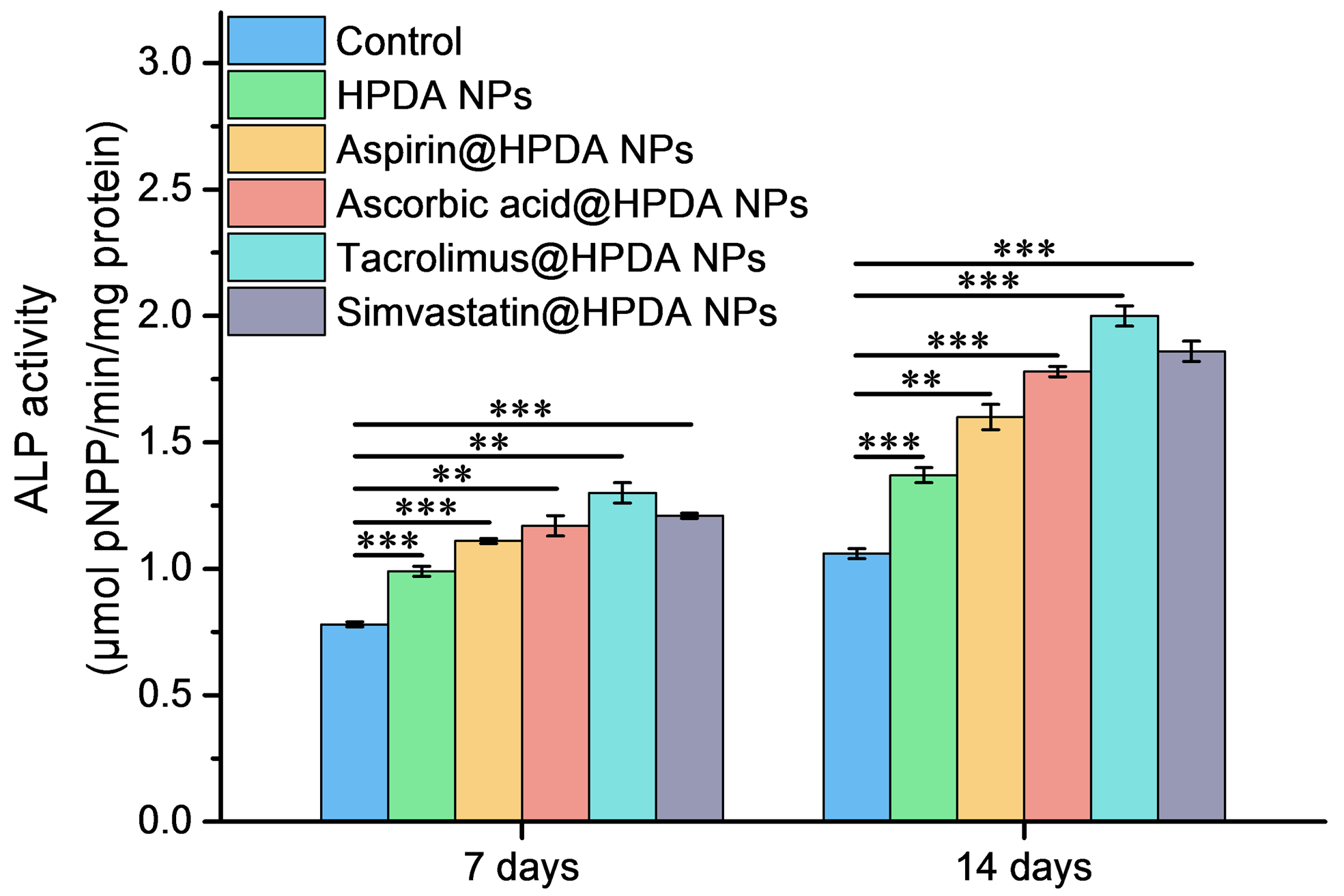


**Figure S8.** Quantitative analyses of ALP activity in 7 and 14 days.


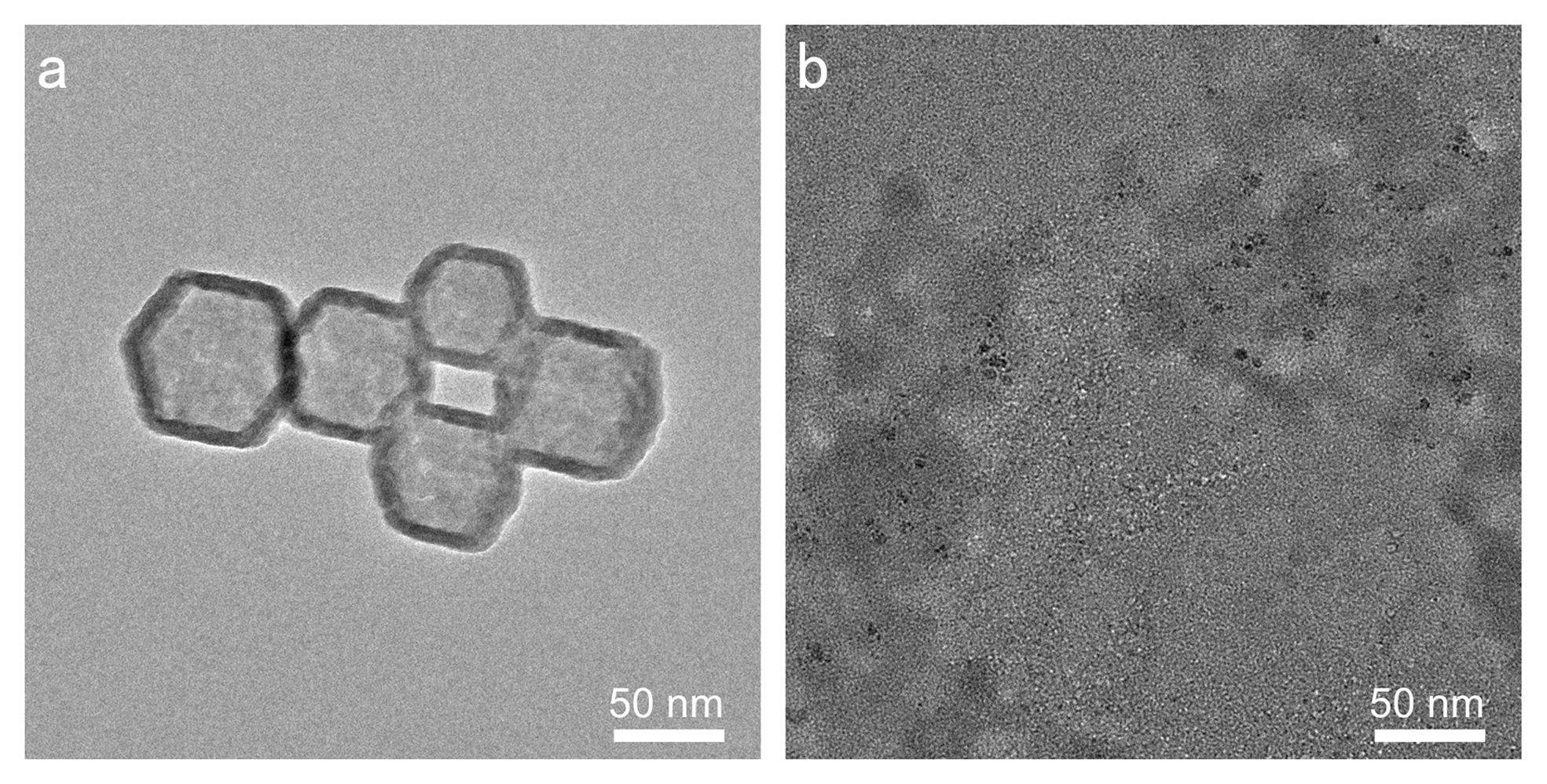


**Figure S9.** (a) TEM images of HPDA NPs. (b) TEM images of HPDA NPs after 4 weeks of subcutaneous incubation in rats.


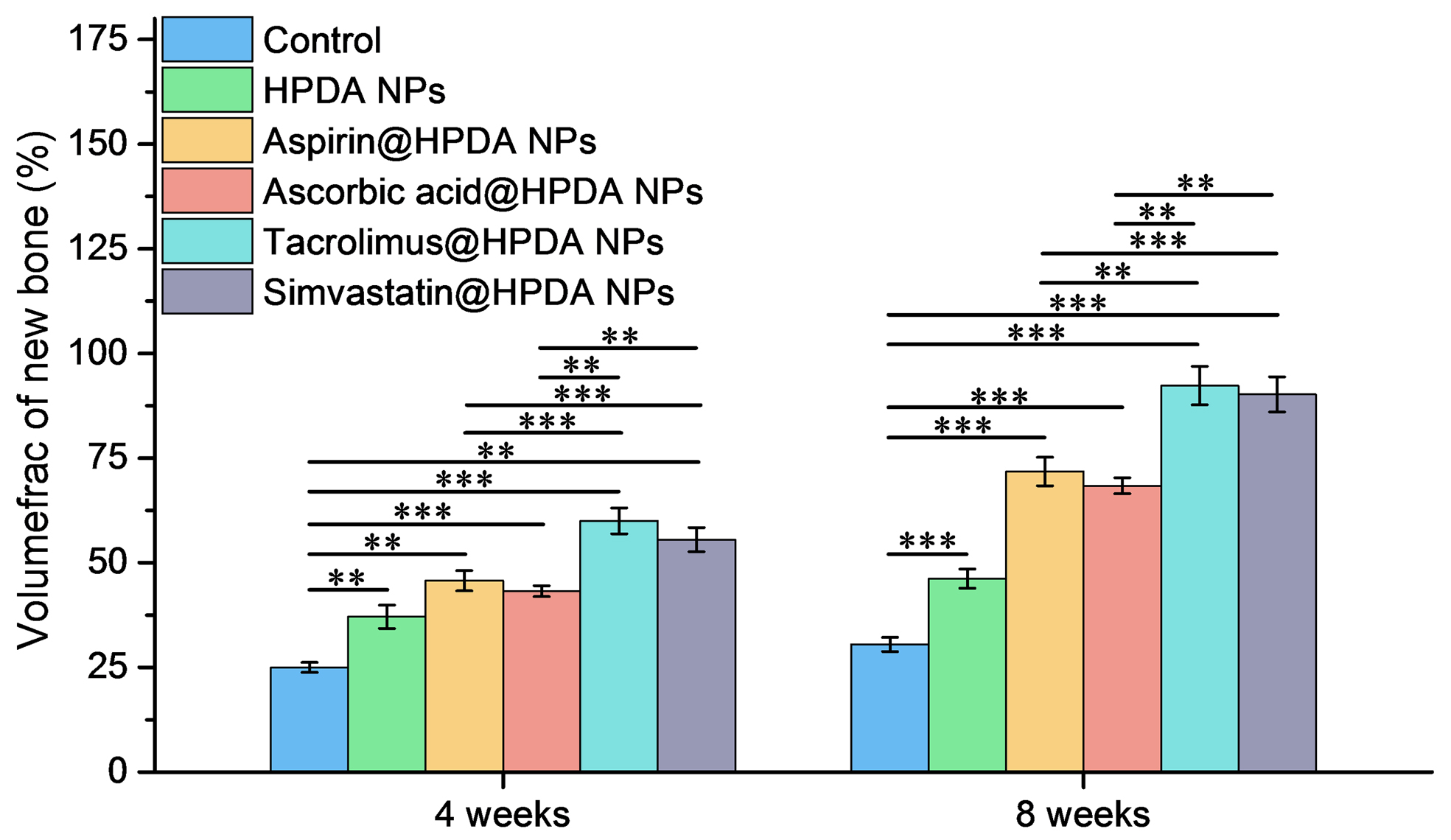


**Figure S10.** Quantitative analyses of Micro-CT data after 4 and 8 weeks post surgery.


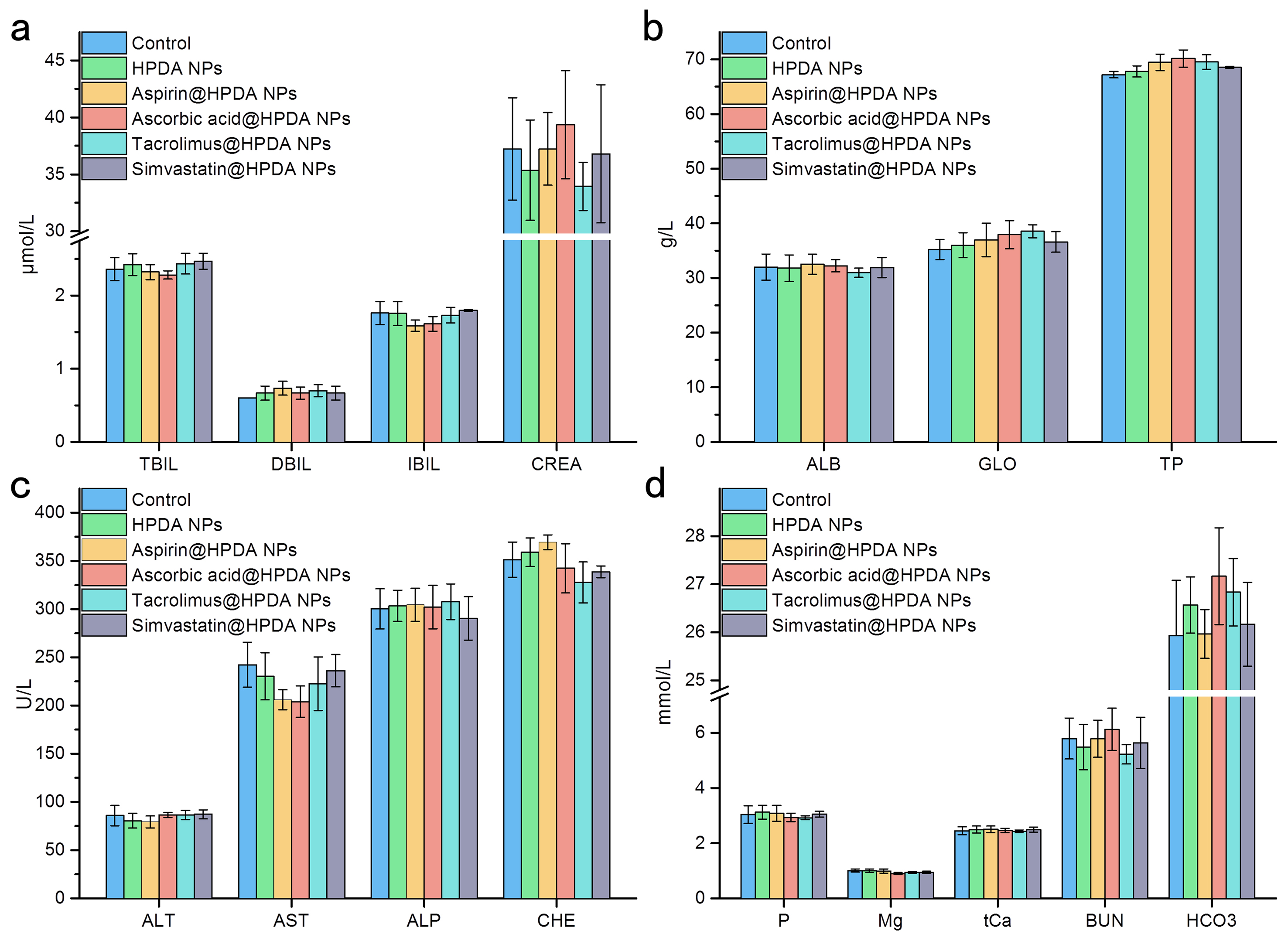


**Figure S11.** Functional evaluations of liver and kidney.


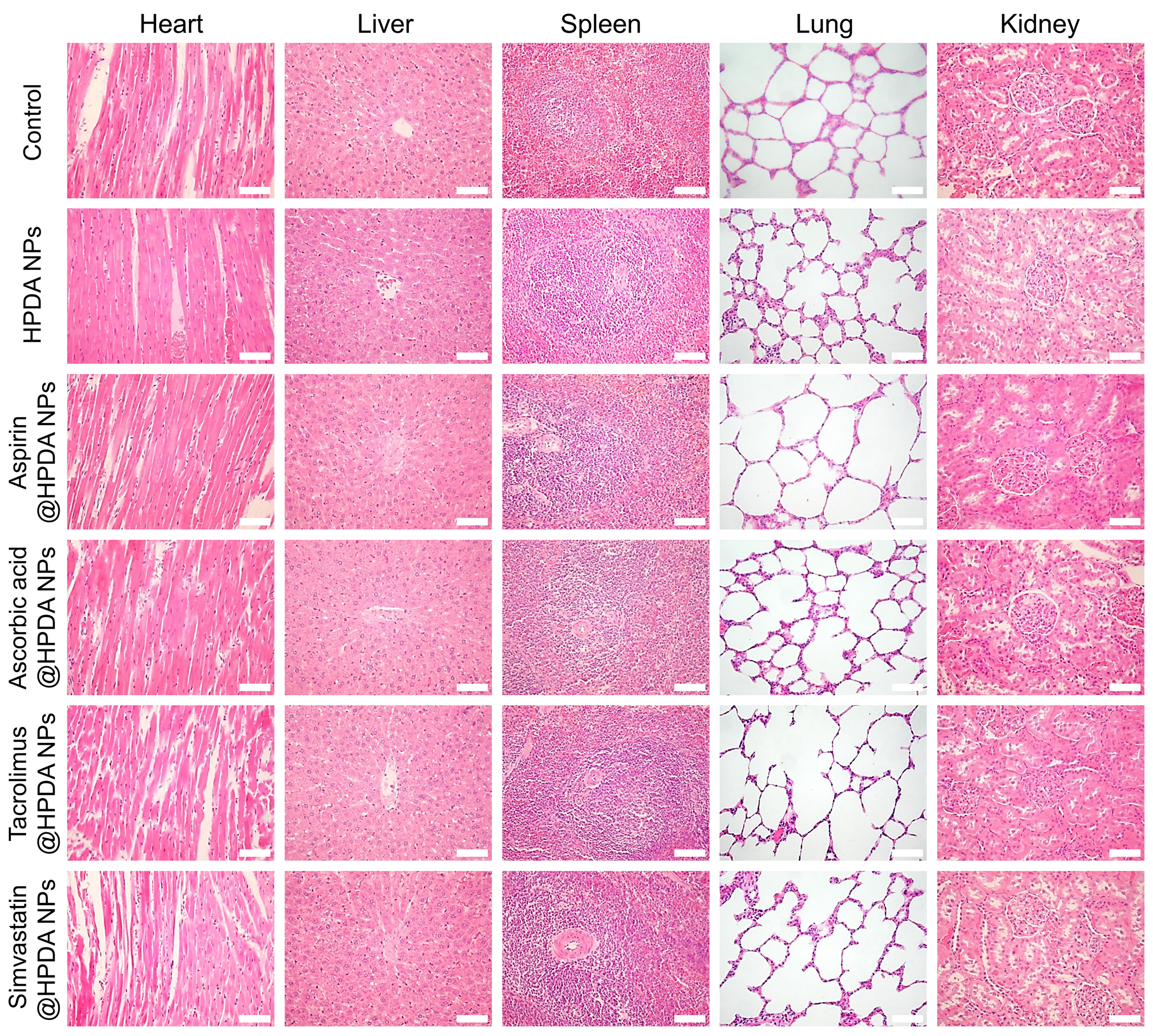


**Figure S12.** H&E stainings of heart, liver, spleen, lung and kidney after 8 weeks post surgery. Scale bar is 100 μm.

**Table S2.** Primer sequences used for qRT-PCR.


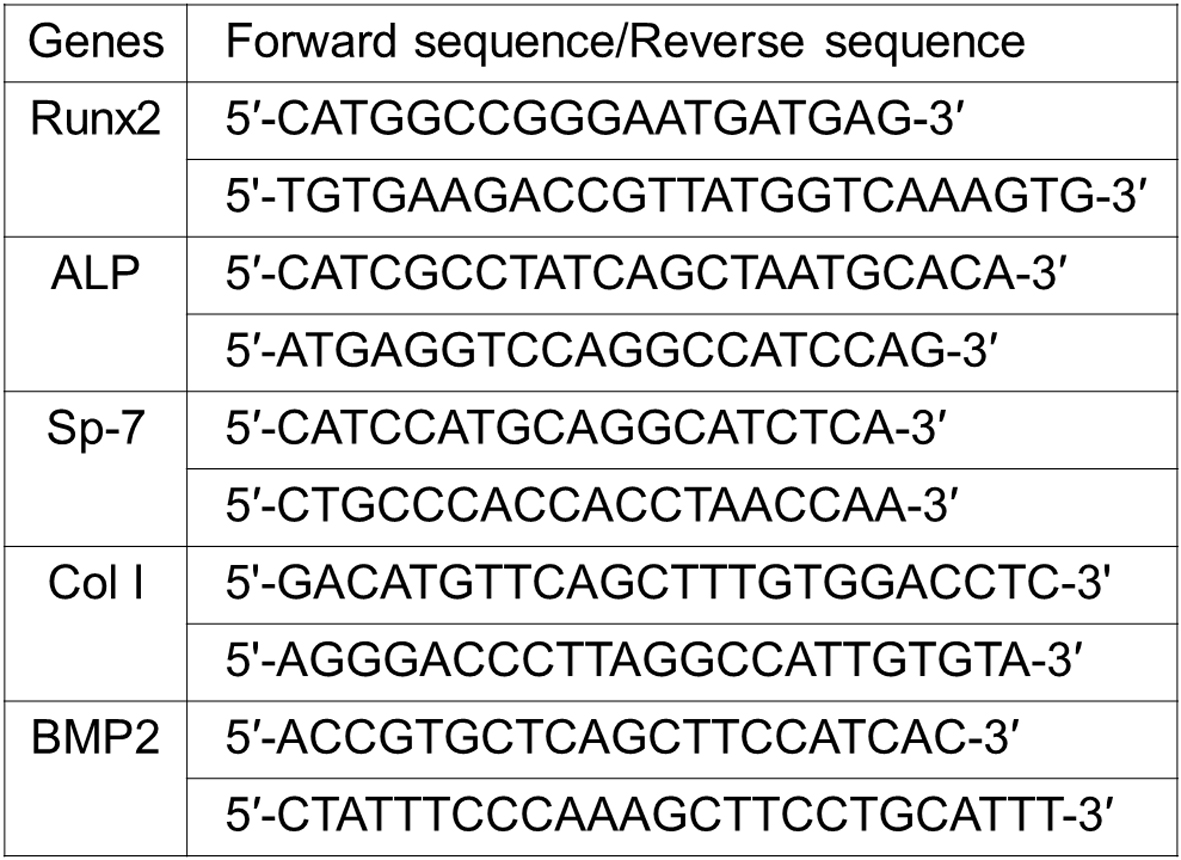

Supplement: Supplementary file 1 — SUPPLEMENTAL MATERIAL [file 41368_2021_132_MOESM1_ESM.doc]
